# Dengvaxia controversy: impact on vaccine hesitancy

**DOI:** 10.7189/jogh.08-020312

**Published:** 2018-12

**Authors:** Khunsha Fatima, Najah Irfan Syed

**Affiliations:** 1 Dow Medical College, Dow University of Health Sciences, Karachi, Pakistan; 2 Bahria University Medical and Dental College, Karachi, Pakistan

Dengue is a viral infection found in tropical and sub-tropical climates worldwide [1]. It is the most rapidly spreading mosquito-borne viral disease [2]. During the past five decades, the global incidence of dengue has risen 30-fold, with the disease now endemic in more than 100 countries [1,2]. So far there is no specific treatment for dengue infection [1].

The quest for a suitable vaccine for dengue has been ongoing for the last six decades [3]. In one such effort, Sanofi, one of the biggest multinational pharmaceutical companies, developed the world’s first dengue vaccine - Dengvaxia. The vaccine is now approved in 19 countries and was used in vaccination campaigns in Philippines, involving more than 800 000 school children [4,5].

**Figure:**
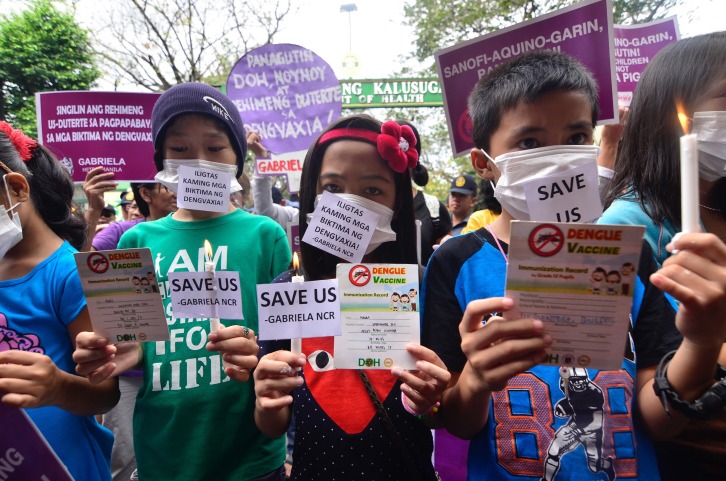
Photo: Gabriela Metro Manila holds protest at DOH main office today over poor government response to Dengvaxia fiasco. Photo by Manila Today (used with permission)

Soon after its authorization, Dengvaxia has now become a subject to controversy following Sanofi’s recent analysis which suggests that the vaccine may put some people at an increased risk of a more severe form of dengue [5]. Dengvaxia was found to reduce the overall risk of severe dengue and hospitalizations due to this disease [4]. This protection, however, was more apparent in those who had a prior history of dengue infection. Sanofi recently discovered that individuals who have never had a dengue infection before pose a significantly higher risk of a more severe form of the disease and hospitalizations with Dengvaxia than if they had not been vaccinated against dengue at all [4].

Considering dengue infection rates reach up to 90% in Philippines, majority of the school children who were inoculated with Dengvaxia will get the protective benefits of the vaccine [5]. This projection, however, means that at least 10% or around 80 000 of those children who do not have a prior history of dengue infection are now at an increased risk of developing severe dengue [6]. Dengvaxia’s sale and distribution has been suspended in Philippines but due to the fear resulting from this controversy, parents are now refusing to vaccinate their children even against vaccine preventable diseases, giving rise to a phenomenon known as Vaccine Hesitancy [7].

“Vaccine hesitancy” refers to delay in acceptance or refusal of safe vaccines despite the availability of vaccination services. Even today, 1 in 5 children worldwide fail to receive routine immunization, and about 1.5 million children die each year of diseases that could be prevented by vaccination [8]. Concern over vaccine safety is one of the most dominant reasons for vaccine hesitancy. Earlier, these concerns were mainly due to widely circulating media reports highlighting a rare occurrence of an adverse reaction to a vaccine, or associating certain disorders to vaccines or their components [9]. Most of these concerns, however, were based on rumors rather than the facts and yet somehow managed to instill fear in the hearts and minds of parents. Unfortunately, the crisis that we are dealing with now is based on results from clinical trials backed up by autopsies linking some deaths to Dengvaxia [6]. These findings may serve as proof against vaccine safety and might have a negative impact on other vaccination programs. With the news being widely circulated through media, this would not just affect Philippines but also other parts of the world.

In light of the above discussion, it is necessary to educate the public regarding safety and success of all other available vaccines through mass education programs, awareness campaigns and conferences to overcome fear and confusion. Media can play a key role in eliminating misconceptions and skepticism resulting from this controversy. Highlighting the positive roles and benefits vaccination campaigns possess may prove to be efficacious as previous vaccination programs have had a tremendous success rate in reducing the mortality and morbidity of various infectious diseases worldwide. It must be emphasized that this particular crisis should not be linked with other vaccines and that the medical sector should be trusted in the formation of newer dengue vaccines. Considering the scale and scope of this issue, all government and non-government health care authorities must work together in efforts to regain parents’ trust. WHO, being a global organization, should play its part in supporting and sustaining public health by helping prevent a drop in vaccination rates and ensuring the acceptability of future vaccines.
